# Definition of irreparable rotator cuff tear: a scoping review of prospective surgical therapeutic trials to evaluate current practice

**DOI:** 10.1186/s12891-023-07067-5

**Published:** 2023-12-08

**Authors:** Fa-Chuan Kuan, Chien-An Shih, Wei-Ren Su, Ausberto Velasquez Garcia, Tomoyuki Kuroiwa, Naoya Iida, Kai-Lan Hsu

**Affiliations:** 1 Department of Orthopaedic Surgery, College of Medicine, National Cheng Kung University Hospital, National Cheng Kung University, 138 Sheng-Li Rd, Tainan, Taiwan; 2 Department of Biomedical Engineering, National Cheng Kung University, Tainan, Taiwan; 3 Skeleton Materials and Bio-compatibility Core Lab, Research Center of Clinical Medicine, College of Medicine, National Cheng Kung University Hospital, National Cheng Kung University, Tainan, Taiwan; 4 Division of Traumatology, National Cheng Kung University Medical Center, Tainan, Taiwan; 5 Department of Orthopedic Surgery, Mayo Clinic, Rochester, MN USA; 6 Department of Orthopedic Surgery, Clinica Universidad de los Andes, Santiago, Chile

**Keywords:** Irreparable rotator cuff tear, Retraction, Patte grade, Fatty degeneration, Tangent sign

## Abstract

**Background:**

The definition of irreparable rotator cuff tear (IRCT) is controversial. This scoping review provides definitions used to describe IRCT in the literature. This scoping review (1) identified criteria used in the definition of IRCT and (2) investigated the current state of those criteria in prospective surgical therapeutic trials.

**Methods:**

This scoping review was conducted in accordance with the Preferred Reporting Items for Systematic Reviews and Meta-Analyses extension for Scoping Reviews. PubMed, Scopus, and Web of Science were searched in March 2023. Studies were screened against predetermined inclusion and exclusion criteria. Criteria regarding clinical symptoms, preoperative images, and intraoperative findings were captured respectively.

**Results:**

A total of 41 prospective studies were eligible for inclusion, and 35 studies (85.4%) defined IRCT. IRCT was defined on the basis of the following main criteria: preoperative image findings (28/35), intraoperative findings (24/35), and symptoms (16/35). With regard to preoperative images, IRCT was mainly defined on the basis of retraction of the tendon in the coronal plane (22/28), the severity of fatty degeneration (19/28), and ruptured tendon number or width of the defect in the sagittal plane (17/28).

**Conclusion:**

This scoping review highlights the lack of a standardized definition for IRCT in clinical practice, with common predictive criteria including a duration of over 6 months, retraction beyond 5 cm, Goutallier grade 3 fatty infiltration, and the rupture of two or more tendons. However, surgeons should apply more than one criterion when examining preoperative images and confirm reparability during surgery. A more objective manner of evaluating intraoperative reparability is necessary.

**Supplementary Information:**

The online version contains supplementary material available at 10.1186/s12891-023-07067-5.

## Introduction

Rotator cuff tears (RCTs) are the most common shoulder disease in patients with shoulder problems, and the reported prevalence is up to 20% in the general population [1]. Among RCTs, irreparable rotator cuff tears (IRCTs) present a significant challenge to orthopedic surgeons because of the high failure rate of repair [2]. Various treatment strategies have been reported, but the optimal therapy remains controversial [3, 4]. This controversy results from both the lack of high-quality comparative studies and the unclear definition of IRCT.

Some surgeons define IRCTs on the basis of their size and the number of tendons involved. Cofield et al. define a tear larger than 5 cm as a massive tear [5], and Gerber et al. describe a massive rotator cuff tear as involving two or more tendons [6]. However, tear size and reparability are not always related. Denard et al. [7] identified that up to 80% of massive RCTs are completely repairable. They indicated that a massive tear displayed in preoperative images is not necessarily irreparable, and irreparable tears are not necessarily large in size.

IRCT can also be defined intraoperatively; Warner et al. [8] define them as involving “the inability to achieve a direct repair of the native tendon to the humerus despite mobilizing the soft tissue.” However, the ability to achieve direct repair is not only determined by the tendon itself, but also related to the quality of debridement and mobilization and even the surgical technique of the surgeon. Furthermore, surgeons are unable to form a definite preoperative plan if reparability is only determined intraoperatively.

Due to the heterogenous and subjective definitions of IRCT in the literature, surgeons may have difficulty in selecting criteria to define IRCT when performing clinical trials. Thus, this scoping review provides different definitions used to describe IRCTs. This review (1) identified criteria used to define IRCT and (2) investigated the current state of those criteria. We focused on prospective clinical research because diagnosis should occur preoperatively or intraoperatively.

## Method

### Search strategy

This scoping review was conducted in accordance with the Preferred Reporting Items for Systematic Reviews and Meta-Analyses extension for Scoping Reviews (PRISMA-ScR) guidelines (**Appendix **1) [9]. PubMed, Scopus, and Web of Science were searched in March 2023 using the following keywords: “(irreparable rotator cuff tear) AND (prospective)” in titles, abstracts, or keyword sections (**Appendix **2). After the database search, the keywords were entered into Google Scholar to identify potentially relevant omitted studies.

### Eligibility criteria

After all publications were identified, duplicates were removed, and study selection was performed by two independent reviewers in two phases. During the first phase, titles and abstracts were reviewed for relevance. In the second phase, full-text articles were examined. A senior author was consulted in cases of disagreement over study inclusion, and these disagreements were resolved by consensus. The references of the included studies were screened using the aforementioned method to prevent the omission of relevant articles.

The selected studies satisfied the following criteria: (1) were prospective clinical studies, (2) included patients who received surgical treatment for IRCT, (3) had IRCT as their main subject, and (4) were written in English.

This systematic review excluded (1) retrospective studies, (2) studies related to diagnosis, nonsurgical treatment, or basic science, letters to editors, systematic reviews or meta-analyses, (3) studies without IRCT as the main subject, (4) studies not written in English, and (5) studies without full-text availability. Studies were assessed for eligibility against the criteria summarized in Table 1.

### Data extraction, analysis, and critical appraisal

Data were gathered from all selected studies by two authors. Data on study design, sample size, surgical methods, and the definition of IRCT were gathered. All recorded definitions were copied verbatim to an Excel database, and the criteria embedded in each definition were extracted; these criteria were as follows. The criteria relating to symptoms were duration of shoulder pain or other specific symptoms. The criteria relating to preoperative included tendon retraction, fatty infiltration, ruptured tendon number, and width of the defect in the sagittal plane. The criterion related to intraoperative findings was tendon irreparable after mobilization. We also recorded whether the criteria were part of the definition or the final confirmation. The criteria were listed separately to allow for quantitative, descriptive statistical analysis.

## Results

### Overview of selected studies

**Fig. 1:**
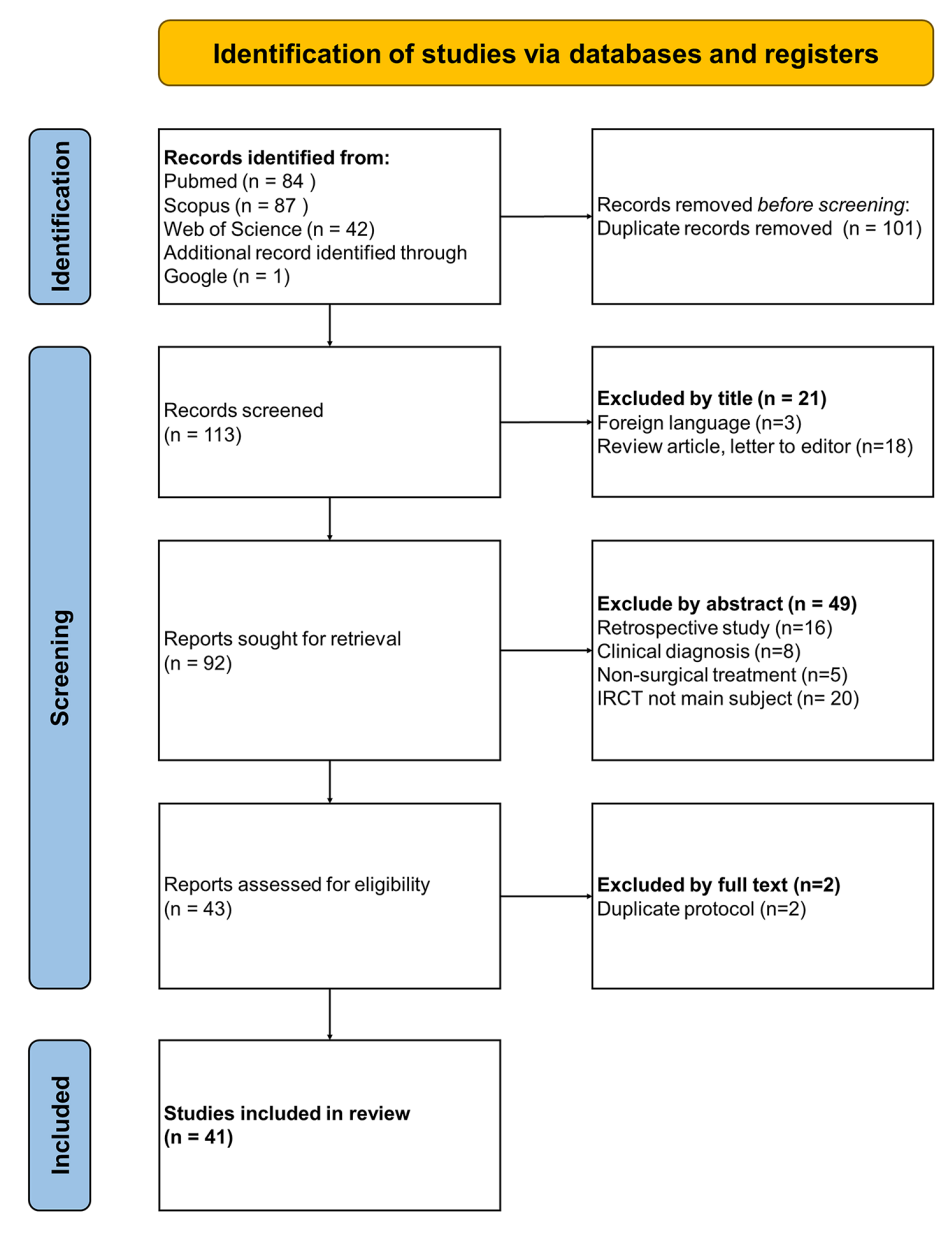
Flowchart of Preferred Reporting Items for Systematic Reviews and Meta-Analyses (PRISMA) guidelines

A total of 113 articles were obtained for review. According to our selection criteria, 41 prospective studies published between 1997 and 2022 were suitable for inclusion **(**Fig. 1**)**. Of the included studies, 32 were case series (78.0%), 6 were nonrandomized control studies (14.6%) and 3 were randomized control studies (7.3%). The reported surgical methods included subacromial spacer placement, partial repair, superior capsule reconstruction, bridging graft, tendon transfer, reverse shoulder arthroplasty (RSA), and other arthroscopic treatment (including debridement, tenodesis, and acromioplasty or tuberoplasty). The characteristics of the included studies are detailed in Table 2.

### Definition of IRCT

In total, 35 studies (85.4%; 35/41) defined IRCT. IRCT was defined on the basis of criteria relating to preoperative image findings (80.0%; 28/35), intraoperative findings (68.6%, 24/35), and symptoms (45.7%, 16/35). In the remaining six eligible studies (14.6%, 6/41), the full-text article did not contain any definition of IRCT, even though each article described IRCT as its main study subject. The details of the three main criteria are listed in Table 3.

**Fig. 2:**
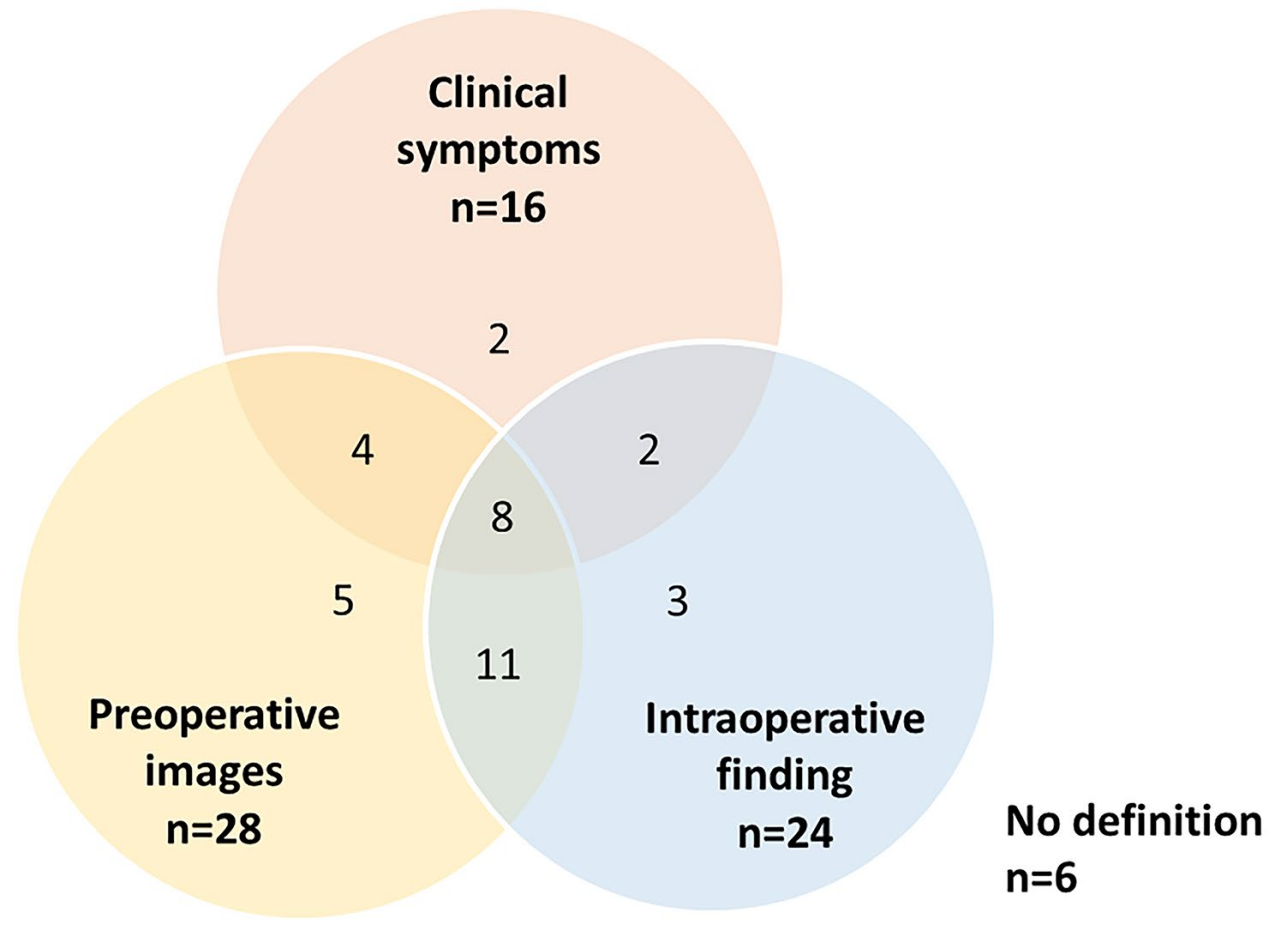
Venn diagram displaying number of studies using three main criteria to define irreparable rotator cuff tear

Of the 35 studies that defined IRCT, 8 (22.9%) used all three main criteria to define IRCT. In 17 (48.6%) studies, two different main criteria were applied, of which the combination of intraoperative findings and preoperative images was most common. In 10 (28.6%) studies, only one main criteria was used, which most commonly related to preoperative images **(**Fig. 2**)**.

Moreover, there was no significant difference (*p* = 0.451) in the number of criteria used between randomized controlled studies, non-randomized controlled studies, and case series **(**Table 4**)**.

### Definition of clinical symptoms

Among the studies providing a definition of IRCT, 45.7% (16/35) applied clinical criteria. The most common clinical feature was symptoms lasting for more than 6 months after conservative treatment followed by symptoms lasting for more than 3 months.

### Definition of preoperative findings

In total, 28 studies used preoperative image findings to define IRCT. In these studies, IRCT was defined on the basis of the following characteristics: retraction of the tendon in the coronal plane (22/28, 78.6%), the severity of fatty degeneration (19/28, 67.9%), and ruptured tendon number or width of the defect in the sagittal plane (17/28, 60.7%). Other characteristics included the severity of muscle atrophy, defect area, and acromiohumeral distance **(**Table 3**)**. The utilization of three primary preoperative image factors did not exhibit significant differences (*p* = 0.787) among randomized controlled studies, non-randomized controlled studies, and case series **(**Table 4**)**.

For retraction of the tendon in the coronal plane, retraction of more than 5 cm was most commonly used for definitions of IRCT. However, retraction of more than 3 cm and Patte grades [10] 3 and 2 were also used. For fatty infiltration, Goutallier grades [11] 3 and higher were most commonly used for definitions, whereas some authors considered tears of Goutallier grades 2 and higher to also be irreparable. With regard to ruptured tendon number or width of the defect in the sagittal plane, more than two ruptured tendons was mostly considered to indicate irreparability.

**Fig. 3:**
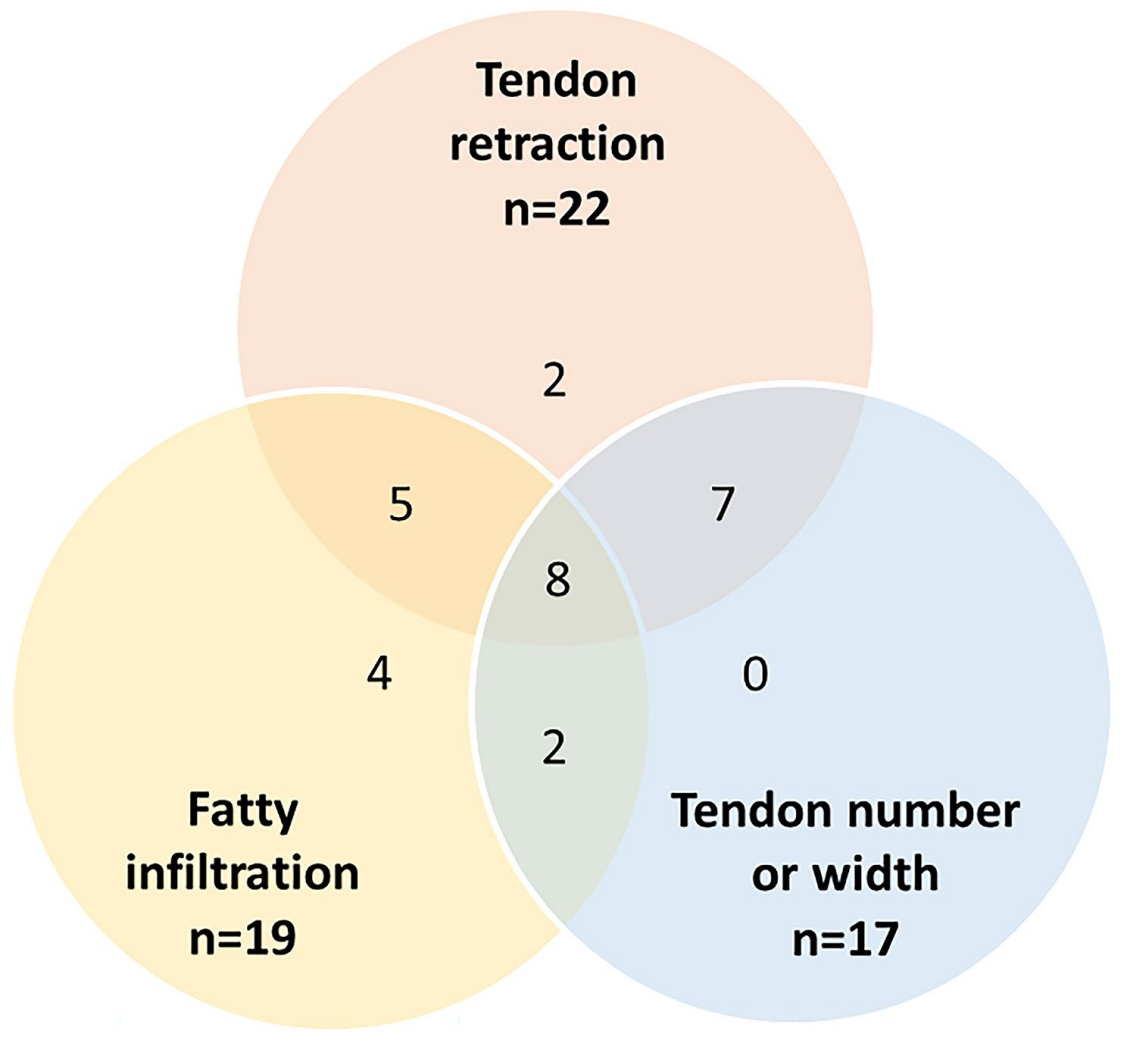
Venn diagram displaying number of studies using three main preoperative image factors to define irreparable rotator cuff tear

Of the 28 studies that used preoperative findings to define IRCT, 8 (28.6%) applied all three main criteria to define IRCT, 14 (50.0%) used two different main criteria, and 6 (21.4%) used only one main criterion **(**Fig. 3**)**.

### Role of intraoperative findings

The criterion “the tendon is irreparable after mobilization” was used to define IRCT in 23 articles, but the role of this criterion differed. In 18 articles, this criterion was used for final confirmation of irreparability, whereas it was part of the definition in the other 6 articles. “Poor quality of the ruptured tendon” was a criterion used in one article.

## Discussion

The present scoping review revealed that clinical symptoms, preoperative images, and intraoperative findings were the three main considerations used to define IRCT. Among preoperative image criteria, tendon retraction, fatty infiltration, and ruptured tendon number or width of defect were primarily used to define IRCT. However, no standardized definition of IRCT is used in clinical trials.

Clinical symptoms are the first criteria used to evaluate patients, but the relationship between clinical symptoms and IRCT is controversial. Although Kuptniratsaikul et al. [12] found that older adulthood (age > 65 years) predicts IRCT, others [13, 14] have not identified this relationship. Pseudoparalysis [15] and lower functional score [13] are also correlated with IRCT. However, these criteria are not widely employed, possibly due to the lack of consensus regarding the definition of pseudoparalysis [16], limited agreement on its diagnosis, and the absence of a clear-cut threshold in the functional score to determine reparability. Anterosuperior escape presents as another robust indicator of IRCT. However, it’s important to note that the treatment for patients exhibiting anterosuperior escape primarily focuses on addressing the associated arthropathy, rather than the rotator cuff itself. As a result, this aspect is seldom discussed in articles pertaining to IRCT. The present review observed that the duration of symptoms was listed in the inclusion criteria for patients with IRCTs in 13 articles, but this was rarely applied as a single criterion. This indicates that this criterion is applied for screening rather than definitive diagnosis.

Preoperative images were used in 80% of the articles to define IRCT, but these criteria were heterogenous. Tendon retraction is correlated with reparability [12, 13, 17, 18] and retear rate [19]; thus, it was applied as a criterion in most articles. However, the retraction cut-off point for determining reparability was controversial. Reparability is indicated by a medial–lateral tear size of smaller than 26 mm per Park et al. [13], 31 mm per Yoo et al. [20], 36 mm per Kuptniratsaikul et al. [12], and 4.2 cm per Kim et al. [21]. Retraction of Patte grade 3 is also a predictor of IRCT [22, 23], but Patte 2 tears were also considered irreparable in two articles. This discrepancy may be attributed to the cutoff Patte stage of IRCT reported by Kim et al. [21], which is 2.5. Consequently, some authors define both Patte 2 and Patte 3 retractions as IRCT. Moreover, it’s possible that they utilized the definition of a large and massive tear, which stipulates that at least one of the two tendons must retract beyond the apex of the humeral head (Patte 2) [24], to categorize it as IRCT. This could be a contributing factor to the variability in the definition. Additionally, examining only one section to assess tendon retraction was insufficient; Guo et al. [25] used a modified Patte classification by using two coronal sections to provide more accurate predictions of reparability.

Fatty infiltration also exhibited significant correlation with tendon reparability, but the cut-off point varied by article. Goutallier grades 2 and 3 are correlated with irreparability [12–14, 21–23]; thus, both were used to define IRCT. However, the cut-off point for fat infiltration may differ between the supraspinatus and infraspinatus muscles [12, 20, 22]. Surgeons should apply this criterion cautiously. Ruptured tendon number, or width of the defect in the sagittal plane, was another predictor of IRCT with reported cut-off points ranging from 22 to 37 mm [12, 13, 20, 21]. However, only one article used a definite value as a criterion. Others evaluated reparability only according to the number of ruptured tendons in the sagittal plane, which is easily assessed in clinical practice.

Factors related to muscle atrophy, such as the tangent sign [15, 21, 22] and muscle occupation ratio [17, 26]) are frequently employed criteria for defining IRCT. It is worth knowing that the tangent sign, which assesses the failure of the supraspinatus to cross a line extending from the superior border of the coracoid process to the superior border of the scapular spine, is a readily performed and reproducible tool with good intraobserver and interobserver reliability [27]. Moreover, it exhibits a high level of accuracy, with a substantial odds ratio for predicting IRCT [28]. In addition to the aforementioned criteria, humeral head upper migration (acromiohumeral interval [13] and inferior glenohumeral distance [21]), and ultrasonography images [29–31] have also been used to evaluate reparability. Surgeons can also use those factors to identify IRCT before surgery when performing prospective clinical studies.

IRCT was defined on the basis of the inability to achieve direct repair after mobilization in 23 of 35 articles. Although this is considered the gold standard for defining repairability in patients with RCTs [15, 22], it was not applied in all articles. This may be because defining irreparability intraoperatively is subjective because no consensus exists, despite the ability of surgeons to intuitively recognize irreparability. To evaluate reparability, repair tension can also be measured. Repair tension is correlated with preoperative retraction and postoperative integrity [32]. Repair tension of more than 30 to 35 N is related to a high retear rate and may not indicate primary repair [33, 34]. Nonetheless, repairability is determined only intraoperatively, not preoperatively, which can complicate surgical decision-making.

Another finding of this scoping review is that more than two factors were applied to define IRCT in approximately 70% of the articles. More than two preoperative image finding factors were used to define IRCT in more than 75% of the articles that included image findings within their criteria. This indicates that surgeons attempt to select patients with relatively strict criteria. Kim et al. [22] and Prasathaporn [35] also identified that the specificity of prediction increases with the application of multiple factors. Moreover, each factor may contribute to prediction of reparability to a different extent. Kim et al. [15] and Park et al. [13] designed a scoring system with different weightings for each factor to predict the reparability of rotator cuffs. The scoring system can also be used to select patients for studies related to IRCT.

The current review has some limitations. First, our aim was to incorporate high-quality publications and gain insight into the criteria established prior to surgery rather than postoperatively. Unfortunately, this exclusion of retrospective studies might have resulted in missing potentially valuable insights from such studies. Second, this was a deliberate choice because our analysis focused solely on the “criteria” presented in the articles, rather than their outcomes. However, this selection may impact the results of this scoping review. Third, the inclusion criteria in each article may have been affected by the surgical methods used. For example, patients who expected to receive RSA may not have been assessed for rotator cuff reparability during operation, and surgeons may have administered debridement only to older patients. Finally, this article primarily addressed the criteria for selecting IRCT patients, but it did not delve into the actual relationship between these criteria and IRCT. Future research endeavors could center on examining the clinical evidence associated with these criteria and assessing their predictive capability for IRCT.

While this scoping review reveals the absence of a standardized definition for IRCT in clinical practice, certain criteria were commonly employed to predict IRCT. These criteria encompass a duration of more than 6 months, retraction exceeding 5 cm, Goutallier grade 3 in fatty infiltration, and the rupture of two or more tendons. However, a more objective manner of evaluating intraoperative reparability is necessary.

**Table 1:** Inclusion and exclusion criteria for study selection

Characteristics	Inclusion	Exclusion
Study availability	Full text is available	Only abstract or title
Study type	Original study	Systematic review, letter to editor
Study contains	Therapeutic IRCT as the main subject	Diagnostic IRCT not the main subject
Study design	Prospective	Retrospective
Language	English	Other than English

IRCT, irreparable rotator cuff tear

**Table 2:** Description of included studies

Author (Year)	Journal/Book	Surgical method	Number of the patient	Definition		
				Clinical symptoms	Preoperative images	Intraoperative findings
***Randomized control study***						
Verma, N. et al. (2022) [36]	J Bone Joint Surg Am	Subacromial spacer	93		V	V
		Partial repair	91			
Ono, Y. et al. (2022) [37]	Arthroscopy	SCR	25		V	V
		BG	25			
Ozturk, B. Y. et al. (2021) [38]	J Shoulder Elbow Surg	Tendon transfer	21	V		
		SCR	21			
***Non-randomized control study***						
Kandeel, A. A. (2023) [39]	Orthop J Sports Med	Partial repair + SCR	15		V	V
		Partial repair + SCR + Tendon transfer	9			
Greiner, S. et al. (2021) [40]	Orthop J Sports Med	SCR	20		V	V
		Partial repair	20			
Cavalier, M. et al. (2018) [41]	Orthop Traumatol Surg Res	Non-operative	71		V	
		Others	26			
		Partial repair	61			
		Tendon transfer	25			
		RSA	35			
Kolk, A. et al. (2018) [42]	Bone Joint J	Tendon transfer	39		V	
Pandey, R. et al(2017) [43]	Shoulder Elbow	Partial repair	13	V	V	V
		Partial repair with BG	13			
Franceschi, F. et al. (2015) [44]	Knee Surg Sports Traumatol Arthrosc	Others	34	V		
		Partial repair	34			
***Case series***						
Dhir, R. et al. (2022) [45]	JSES Reviews, Reports, and Techniques	Subacromial spacer	4		V	
Gbejuade, H. et al. (2022) [46]	Shoulder Elbow	SCR	17	V	V	V
Shin, S. J. et al. (2022) [47]	Arthroscopy	SCR	21	V		V
Reinares, F. et al. (2022) [48]	Eur J Orthop Surg Traumatol	Tendon transfer	15	V	V	
Liao, Y. T. et al. (2022) [49]	J Orthop Surg Res	SCR	19			
Haque, A. et al. (2021) [50]	J Clin Orthop Trauma	BG	22	V	V	V
Familiari, F. et al. (2021) [51]	Arthroscopy	Subacromial spacer	51	V	V	
Zafra, M. et al. (2021) [52]	J Orthop	SCR	5		V	V
Lacheta, L. et al. (2020) [53]	Arthroscopy	SCR	22		V	V
Ulstrup, A. et al. (2020) [54]	JSES Int	SCR	13	V	V	V
Azevedo, C. I. C. et al. (2020) [55]	Am J Sports Med	SCR	22			V
Polacek, M. (2019) [56]	Arthrosc Sports Med Rehabil	SCR	19	V	V	
Mirzaee, F. et al. (2019) [57]	Arch Bone Jt Surg	Others	12			
Valenti, P. et al. (2019) [58]	Int Orthop	Tendon transfer	31		V	V
Matsen, F. A., 3rd et al. (2019) [59]	Int Orthop	Others	40			V
Iban, M. A. R. et al. (2018) [60]	Knee Surgery Sports Traumatology Arthroscopy	Subacromial spacer	15	V	V	V
Yallapragada, R. K. et al. (2018) [61]	J Orthop	Subacromial spacer	14		V	V
Piekaar, R. S. M. et al(2018) [62]	Musculoskelet Surg	Subacromial spacer	44			
Ruiz Iban, M. A. et al(2018) [63]	Knee Surg Sports Traumatol Arthrosc	Subacromial spacer	15	V	V	V
Denard, P. J. et al. (2018) [64]	Arthroscopy	SCR	59			
Hirahara, A. M. et al. (2017) [65]	Am J Orthop (Belle Mead NJ)	SCR	9			
Kany, J. et al. (2016) [66]	Eur J Orthop Surg Traumatol	Tendon transfer	5		V	
Grimberg, J. et al. (2015) [67]	Arthroscopy	Tendon transfer	54		V	V
Modi A, et al. (2013) [68]	Shoulder Elbow	BG	61		V	V
Senekovic, V. et al. (2013) [69]	Eur J Orthop Surg Traumatol	Subacromial spacer	20	V		V
Gupta, A. K. et al. (2013) [70]	Am J Sports Med	BG	26	V	V	V
Henseler, J. F. et al. (2013) [71]	Bone Joint J	Tendon transfer	28		V	
Gupta, A. K. et al. (2012) [72]	Am J Sports Med	BG	24	V	V	V
John, M. et al. (2010) [73]	Int Orthop	RSA	15	V	V	
Gerber, C. et al. (2006) [74]	J Bone Joint Surg Am	Tendon transfer	14			
Malkani, A. L. et al. (2004) [75]	Clin Orthop Relat Res	Tendon transfer	18			V
Gartsman, G. M. (1997) [76]	J Bone Joint Surg Am	Others	33		V	V

BG, bridging grafting; RSA, reverse shoulder arthroplasty; SCR, superior capsule reconstruction

**Table 3:** Definition of irreparable rotator cuff tears in prospective surgical therapeutic trials

Classification	Characteristics	Detail	Number of articles	Reference
**Symptoms**	Duration of symptoms	≥ 6 months	13	[38, 43, 44, 46–48, 51, 56, 60, 63, 69, 70, 72]
		≥ 3 months	2	[50, 54]
		Pseudoparalysis	1	[73]
**Images**	Tendon retraction	Retraction ≥ 5 cm	9	[36, 37, 43, 46, 50, 56, 70, 72, 76]
		Retraction ≥ 3 cm	5	[51, 60, 63, 68, 71]
		≥ Patte grade 3	6	[40, 45, 48, 53, 61, 67]
		≥ Patte grade 2	2	[39, 42]
	Fatty infiltration	≥ Goutallier grade 3 (or infiltration ≥ 50%)	11	[39, 40, 45, 51–54, 60, 61, 63, 67]
		≥ Goutallier grade 2 (or ≥ Fuchs grade 2)	5	[41, 42, 48, 58, 73]
		Goutallier grade 1–3	1	[70]
		Goutallier grade 4	2	[56, 66]
	Ruptured tendon number or width of the defect in sagittal plain	≥ 2 tendons	12	[36, 37, 41, 42, 46, 48, 50, 58, 67, 68, 70, 76]
		Anterior to posterior defect ≥ 5 cm	1	[39]
		Anterior to posterior defect ≥ 3 cm	3	[51, 60, 63]
		≥ Bayne and Bateman grade 3	1	[40]
	Muscle atrophy	≥ Thomazeau grade 3	2	[39, 53]
		Positive tangent sign	1	[54]
	Other – Defect area	Davidson group 3	1	[42]
	Other – AH interval	≤ 6 mm AHD	1	[52]
**Intraoperative finding**	Irreparable after mobilization	Act as final conform	18	[36, 37, 40, 43, 46, 47, 50, 52, 53, 55, 59–61, 68–70, 75, 76]
		Part of the criteria	5	[54, 58, 63, 67, 72]
	Poor tendon quality	Part of the criteria	1	[39]

AHD, acromial humeral distance

**Table 4:** The number of used three main criteria and three main preoperative image factors in predicting irreparable rotator cuff tears between different level articles

	Randomized control study	Non-randomized control study	Case series	P-value
Number of used three main criteria				0.451
0	0	0	6	
1	1	3	6	
2	2	2	13	
3	0	1	7	
Number of used three main preoperative image factors				0.787
0	1	1	11	
1	0	1	5	
2	2	2	10	
3	0	2	6	

### Electronic supplementary material

Below is the link to the electronic supplementary material.

Appendix 1

Appendix 2

## Data Availability

All data included in this study are available upon request by contact with the corresponding author on reasonable request.
