# Supplementary material for: Definition of irreparable rotator cuff tear: a scoping review of prospective surgical therapeutic trials to evaluate current practice
Source: BMC Musculoskelet Disord. 2023 Dec 8;24:952. doi: 10.1186/s12891-023-07067-5 (PMC10704799; doi:10.1186/s12891-023-07067-5)
Supplement: Supplementary file 2 — Appendix 2 [file 12891_2023_7067_MOESM2_ESM.docx]

**Appendix 2: Detail Search strategy and results**

**Search in Pubmed**

| Strategy | (irreparable rotator cuff tears) AND (prospective) in all field |
| --- | --- |
| Result (n=84) | {Agout, 2018 #16414;Azevedo, 2020 #16394;Badman, 2020 #16395;Beraldo, 2022 #16369;Boileau, 2002 #16444;Boutsiadis, 2017 #16418;Carbone, 2018 #16409;Cavalier, 2018 #16411;Collin, 2015 #16427;Cox, 2023 #16378;Cusano, 2022 #16375;Denard, 2018 #16420;Diaz Balzani, 2022 #16365;Elkins, 2019 #16402;Ellman, 1993 #16446;Familiari, 2021 #16393;Fang, 2019 #16401;Franceschi, 2015 #16435;Freislederer, 2022 #16383;Gartsman, 1997 #16445;Gartsman, 2013 #16430;Gbejuade, 2022 #16372;Gerber, 2006 #16441;Greiner, 2021 #16389;Grimberg, 2015 #16428;Gumina, 2013 #16431;Gupta, 2012 #16437;Gupta, 2013 #16433;Haque, 2021 #16380;Henseler, 2017 #16421;Henseler, 2013 #16432;Hirahara, 2017 #16419;Hohmann, 2021 #16382;Hughes, 2022 #16371;Hughes, 2021 #16388;Kandeel, 2023 #16364;Kolk, 2018 #16417;Lacheta, 2020 #16398;Ladermann, 2018 #16416;Ladermann, 2021 #16381;Lee, 2020 #16391;Levy, 2008 #16440;Liao, 2022 #16368;Lichtenberg, 2012 #16436;Longo, 2011 #16438;Maillot, 2018 #16415;Malahias, 2019 #16407;Malkani, 2004 #16443;Matsen, 2019 #16406;Matsumura, 2017 #16423;Mirzaee, 2019 #16403;Mirzayan, 2023 #16367;Modi, 2022 #16376;Muench, 2020 #16399;Mulieri, 2010 #16439;O'Holleran, 2005 #16442;Ono, 2022 #16373;Osti, 2021 #16384;Ozturk, 2021 #16390;Pandey, 2017 #16422;Piekaar, 2020 #16404;Polacek, 2019 #16396;Postacchini, 1992 #16447;Prinja, 2021 #16386;Reinares, 2022 #16385;Rosales-Varo, 2019 #16408;Ruiz Iban, 2018 #16413;Sandhu, 2023 #16370;Seker, 2018 #16410;Senekovic, 2013 #16434;Senekovic, 2017 #16425;Shin, 2022 #16379;Srikumaran, 2023 #16366;Stewart, 2019 #16400;Thompson, 2020 #16405;Tse, 2016 #16426;Ulstrup, 2020 #16392;Valenti, 2015 #16429;Veen, 2020 #16397;Verma, 2022 #16374;Viswanath, 2021 #16387;Yallapragada, 2018 #16412;Yian, 2017 #16424;Zafra, 2021 #16377} |

**Search in Scopus**

| Strategy | ( TITLE-ABS-KEY ( irreparable AND rotator AND cuff AND tear ) AND TITLE-ABS-KEY ( prospective ) ) |
| --- | --- |
| Result (n=87) | {, 2021 #15713;Agout, 2018 #15734;Agout, 2018 #15735;Azevedo, 2020 #15717;Badman, 2020 #15718;Beraldo, 2022 #15692;Boileau, 2002 #15769;Boutsiadis, 2017 #15745;Burkhart, 2020 #15722;Calek, 2022 #15696;Carbone, 2018 #15736;Cavalier, 2018 #15731;Collin, 2015 #15754;Cox, 2023 #15687;Cusano, 2022 #15695;Denard, 2018 #15743;Dhir, 2022 #15693;Diaz Balzani, 2022 #15689;Dukan, 2020 #15719;Elkins, 2019 #15727;Ellman, 1993 #15770;Familiari, 2021 #15712;Fang, 2019 #15725;Franceschi, 2015 #15755;Garofalo, 2023 #15688;Gartsman, 2013 #15756;Gilat, 2021 #15711;Grimberg, 2015 #15752;Gumina, 2013 #15761;Gupta, 2012 #15764;Gupta, 2013 #15760;Haque, 2021 #15705;Henseler, 2017 #15744;Henseler, 2013 #15762;Hohmann, 2021 #15707;Hughes, 2022 #15700;Hughes, 2021 #15709;John, 2010 #15767;Kandeel, 2023 #15686;Kane, 2022 #15699;Kany, 2016 #15750;Khan, 2011 #15765;Kolk, 2018 #15740;Lacheta, 2020 #15720;Lädermann, 2018 #15739;Lädermann, 2021 #15703;Lee, 2022 #15701;Levy, 2008 #15768;Liao, 2022 #15690;Lichtenberg, 2012 #15763;Longo, 2011 #15766;Maillot, 2018 #15738;Matsen, 2019 #15729;Matsumura, 2017 #15746;Mirzaee, 2019 #15728;Modi, 2022 #15702;Modi, 2013 #15758;Morris, 2015 #15751;Noyes, 2018 #15741;Ono, 2022 #15691;Osti, 2021 #15706;Ozturk, 2021 #15708;Piekaar, 2018 #15732;Piekaar, 2020 #15716;Polacek, 2019 #15724;Postacchini, 1992 #15771;Reinares, 2022 #15697;Revathi, 2018 #15742;Ronquillo, 2013 #15757;Rosales-Varo, 2019 #15730;Ruiz Ibán, 2018 #15733;Sebastia-Forcada, 2020 #15715;Senekovic, 2013 #15759;Senekovic, 2017 #15749;Shields, 2017 #15748;Shin, 2022 #15698;Srikumaran, 2023 #15685;Stewart, 2019 #15726;Thompson, 2020 #15723;Ulstrup, 2020 #15714;Valenti, 2015 #15753;Veen, 2020 #15721;Verma, 2022 #15694;Viswanath, 2021 #15710;Yallapragada, 2018 #15737;Yian, 2017 #15747;Zafra, 2021 #15704} |

**Search in WEB of science**

| Strategy | (irreparable rotator cuff tears) AND (prospective) IN TITLE-ABS-KEY |
| --- | --- |
| Result (n=42) | {Agout, 2018 #16467;Azevedo, 2020 #16459;Bailey, 2019 #16466;Cavalier, 2018 #16469;Davidson, 2010 #16485;Edwards, 2006 #16486;Ellman, 1993 #16489;Franceschi, 2015 #16480;Gartsman, 2013 #16481;Grimberg, 2015 #16478;Gupta, 2012 #16483;Henry, 2015 #16477;Henseler, 2017 #16474;Henseler, 2013 #16482;Hohmann, 2021 #16453;Hughes, 2022 #16450;Hughes, 2021 #16456;Iban, 2018 #16468;Kandeel, 2023 #16448;Kessler, 2003 #16487;Kolk, 2018 #16473;Ladermann, 2018 #16472;Lee, 2022 #16451;Liao, 2022 #16449;Longo, 2011 #16484;Maillot, 2018 #16471;Matsen, 2019 #16464;Mirzaee, 2019 #16465;Modi, 2022 #16452;Osti, 2021 #16454;Osti, 2020 #16460;Ozturk, 2021 #16455;Reinares, 2022 #16457;Sebastia-Forcada, 2020 #16458;Senekovic, 2017 #16476;Shields, 2017 #16475;Soyer, 2003 #16488;Stewart, 2019 #16462;Thompson, 2020 #16463;Valenti, 2015 #16479;Veen, 2020 #16461;Yallapragada, 2018 #16470} |
